# Serum BDNF levels as a potential prognostic marker for functional recovery in stroke: Preliminary findings from a prospective observational study

**DOI:** 10.1371/journal.pone.0343929

**Published:** 2026-02-27

**Authors:** Seyoung Shin, Heegoo Kim, Dae Hyun Kim, Won Hyuk Chang

**Affiliations:** 1 Department of Rehabilitation Medicine, CHA Bundang Medical Center, CHA University School of Medicine, Seongnam, Republic of Korea; 2 Department of Physical and Rehabilitation Medicine, Samsung Medical Center, Sungkyunkwan University School of Medicine, Seoul, Republic of Korea; 3 Department of Health Science and Technology, SAIHST, Sungkyunkwan University, Seoul, Republic of Korea; Beijing University of Chinese Medicine Affiliated Dongzhimen Hospital, JAPAN

## Abstract

Brain-derived neurotrophic factor (BDNF) crosses the blood-brain barrier and may serve as a marker of neuroplasticity. This study evaluated whether serum levels of mature BDNF, proBDNF, and matrix metalloproteinase-9 (MMP-9) can predict functional recovery after stroke. In this prospective observational study, 93 patients with unilateral stroke and motor impairment were recruited. Clinical, and demographic data, as well as serum levels of mature BDNF, proBDNF, and MMP-9 were collected. Functional assessments measuring stroke severity, cognition, motor function, balance, and mood were conducted at three timepoints: after acute care (T0), 2 weeks post-rehabilitation (T1), and 3 months post-onset (T2). Mature BDNF significantly decreased from T0 to T2 (p = 0.003), while proBDNF remained stable. MMP-9 declined consistently across timepoints (p < 0.001). MMP-9 levels at baseline differed by BDNF genotype (p < 0.05). However, none of the biomarkers independently predicted functional recovery. Functional outcomes improved significantly over time (p < 0.001), with baseline functional scores being the strongest predictors at T1 and T2. Although these biomarkers were not independent predictors of recovery, their longitudinal trajectories may reflect underlying neurobiological recovery mechanisms during rehabilitation, although their prognostic utility remains inconclusive.

## Introduction

The brain undergoes self-repair after injury, such as stroke, leading to functional recovery. The mechanisms by which the brain achieves functional recovery are referred to as neuroplasticity, and they include processes such as synaptic plasticity, dendritic outgrowth, and axonal sprouting [1,2]. Identifying objective neuroplasticity markers through blood sampling can help predict stroke prognosis and support the development of neuroplasticity-enhancing treatments [3]. Previous studies have shown that the brain-derived neurotrophic factor (BDNF) is an important neuroprotective factor [4]. However, most studies have measured serum BDNF levels only once in patients with stroke.[5] In the study by Sobrino et al. [6], serum BDNF levels significantly decreased from the acute phase to 3 and 12 months poststroke in 445 patients. Furthermore, serum BDNF levels were not associated with infarct volume or functional outcomes. Although their study focused on patients in the acute stroke phase, they did not specify whether these patients received rehabilitation therapy.

Serum BDNF levels can be increased by environmental factors, particularly active exercise [7]. proBDNF is a precursor of mature BDNF. Mature BDNF promotes neuronal survival, growth, and synaptic plasticity, supporting learning and memory. Meanwhile, proBDNF is associated with opposite effects, such as promoting cell apoptosis and synaptic pruning. ProBDNF is converted to mature BDNF through proteolytic cleavage by enzymes or matrix metalloproteinases [4]. Measuring serum levels of mature BDNF, proBDNF, and matrix metalloproteinase-9 (MMP-9) can help elucidate the relationships among these biomolecules and assess their potential as biomarkers. The BDNF genotype is a common single-nucleotide polymorphism (SNP) in the pro-region of the *BDNF* gene, where a G-to-A substitution leads to a valine-to-methionine change (Val66Met). Importantly, it can also influence serum levels of mature BDNF, proBDNF, and MMP-9. BDNF has been linked to increased vulnerability, prevalence, and specific clinical characteristics of various neurodegenerative diseases [8].

This study aimed to examine the serial changes in serum levels of mature BDNF, proBDNF, and MMP-9 during the early subacute stroke phase in patients undergoing comprehensive rehabilitation. Unlike previous studies that measured serum BDNF or MMP-9 levels only once during the acute phase, our study longitudinally tracked these biomarkers throughout the subacute phase under standardized rehabilitation. This design allowed us to capture dynamic neuroplastic processes that occur during active recovery, providing clinical insight beyond static prognostic models.

## Materials and methods

### Study design and patients

This prospective, observational, non-interventional study evaluated a stroke patient cohort hospitalized at Samsung Medical Center. The inclusion criteria were as follows: 1) unilateral stroke, 2) admission to the rehabilitation department within 1 month of stroke onset, and 3) mild to severe motor impairment at the time of transfer to the rehabilitation department. The exclusion criteria were as follows: 1) progressive or unstable stroke; 2) pre-existing and active major neurological or psychiatric diseases; 3) advanced liver, kidney, cardiac, or pulmonary disease; 4) terminal medical conditions with a life expectancy of <1 year, and 5) pregnancy or lactation.

All patients underwent prospective serological and functional assessments at 3 time points: upon transfer to the rehabilitation department after the completion of acute stroke care (T0), 2 weeks after comprehensive rehabilitation (T1), and 3 months post-stroke onset (T2). The standardized inpatient rehabilitation program consisted of 2 hours of physical therapy and 1 hour of occupational therapy daily on weekdays from T0 to T1. Physical therapy included gait training, strengthening exercises, balance training, and task-oriented functional mobility training. Occupational therapy consisted of upper-limb functional training, ADL-focused exercises, cognitive training and fine-motor coordination tasks. Thereafter, the patients participated in either a standardized outpatient rehabilitation program that consisted of 1 hour of physical therapy and 30 minutes of occupational therapy 3 days per week or self-exercise at home until T2.

Written informed consent was obtained from all patients before their inclusion in the study, and the study protocol was approved by the Institutional Review Board (SMC 2016-08-059-002). This study was registered at ClinicalTrials.gov (Identifier: NCT03164798) under the title ‘Serum BDNF Role as a Biomarker for Stroke Rehabilitation’, sponsored by Samsung Medical Center. Recruitment commenced on 1 February 2017 and the estimated completion date was 31 December 2019.

### Data collection

Data on demographic information (age, sex, smoking, and alcohol history) and clinical factors, such as stroke information (side, stroke type, and onset), underlying diseases, body mass index, and BDNF genotype at T0, were collected. Baseline brain MRI performed during acute stroke care was reviewed to confirm stroke diagnosis and classification; however, no standardized quantitative imaging variables were extracted for the present analyses.

Blood samples were collected at T0, T1, and T2. Serum biomarker analysis was conducted using enzyme-linked immunosorbent assay (ELISA). Serum levels of mature BDNF, proBDNF, and matrix metalloproteinase-9 (MMP-9) were measured using commercially available enzyme-linked immunosorbent assay kits (mature BDNF and proBDNF: Adipo Bioscience, Santa Clara, CA, USA; MMP-9: R&D Systems, Minneapolis, MN, USA). Optical density was measured using an automated microplate reader (Emax; Molecular Devices, Sunnyvale, CA, USA), and all assays were performed in accordance with the manufacturers’ instructions. To minimize assay-related variability, serum samples were stored at −80 °C until analysis and were handled using standardized procedures.

Given that outliers were observed, the interquartile range (IQR) method was used to identify and remove them and ensure data integrity [9]. Specifically, any data points that fell outside the range defined by 1.5 times the IQR below the first quartile (Q1) or above the third quartile (Q3) were considered outliers and were excluded from the subsequent analysis (S1 Table).

The presence of the BDNF Val66Met polymorphism at T0 was also assessed. For BDNF genotyping, whole blood was first collected in ethylenediaminetetraacetic acid tubes. Next, genomic DNA was isolated from peripheral blood leukocytes according to a standard proteinase-K RNase digestion procedure followed by phenol-chloroform extraction. Finally, the BDNF Val66Met polymorphism was genotyped via polymerase chain reaction-restriction fragment length polymorphism. The BDNF genotype was classified as Val/Val, Val/Met, or Met and entered as an ordinal predictor according to the number of Met alleles (Val/Val = 0; Val/Met = 1; Met/Met = 2).

### Functional outcome measures

Functional assessments were performed at each time point using the National Institutes of Health Stroke Scale (NIHSS) for stroke severity [10], Korean Mini-Mental State Examination (K-MMSE) for cognitive function, [11] Fugl-Meyer Assessment (FMA) for motor impairment [12], Berg Balance Scale (BBS) for balance function, [13] and Geriatric Depressive Scale-short form (GDS-SF) for depressive mood [14]. Licensed occupational and physical therapists who completed a designated training program conducted the face-to-face functional assessments.

### Statistical analysis

Descriptive statistics were used for baseline patient characteristics, serum biomarker levels, and functional status. Numerical variables are summarized as means and standard deviations or as medians and IQRs according to the normality of their distribution. Categorical variables are presented as percentages. The significance of the changes in serum biomarker levels or functional status between time points was assessed using either paired t-tests or Wilcoxon signed-rank tests, depending on the normality of data distribution. Bonferroni correction was applied for multiple comparisons of repeated measurements. To investigate potential differences in serum biomarker concentrations and functional status according to the BDNF genotype, comparisons were performed among patients with the Val/Val, Val/Met, and Met/Met genotypes. Furthermore, serum biomarker levels were compared among the three groups using analysis of variance with covariates. The covariate adjusted for included sex, age, initial stroke severity (NIHSS score at T0), and stroke type, which were chosen by the physicians.

Independent influencing factors of each functional outcome were identified using multivariate linear regression analysis. First, univariate linear regression analysis was conducted to investigate the relationship between potential influencing factors, including serum biomarker levels at T0 and functional status at the early (T1) and late subacute phases (T2). Variables exhibiting univariate associations (i.e., with p-values <0.20) were included in the multivariate model. Because this was an observational cohort without treatment allocation, a traditional intention-to-treat framework was not applicable. Instead, each time point was analyzed using an available-case approach, in which all patients who had data for a given assessment (T0, T1, or T2) were included in the corresponding analysis. All statistical analyses were performed using R (version 4.0.3; R Foundation for Statistical Computing, Vienna, Austria) and GraphPad Prism (version 10.0; GraphPad Software, San Diego, CA, USA). Statistical significance was set at p < 0.05.

## Results

### Patient characteristics

**Fig 1:**
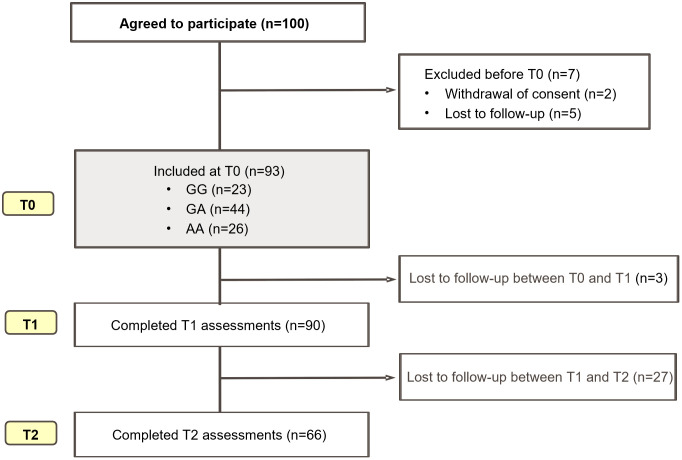
Flow diagram of patient enrollment, exclusions, and follow-up across study time points (T0–T2). T0 indicates transfer to the rehabilitation department after completion of acute stroke care; T1, 2 weeks after comprehensive rehabilitation; and T2, 3 months after stroke onset.

A total of 100 patients initially consented to participate in the study. Before the T0 assessment was initiated, 2 patients withdrew consent and 5 were lost to follow-up, resulting in 93 patients included at T0. Between T0 and T1, 3 additional patients were lost to follow-up, and 90 patients completed the T1 assessment. By T2, a total of 27 patients had been lost to follow-up, and 66 patients completed the T2 assessment (Fig 1.). Because individual participants contributed data at different time points (e.g., some had only T0 and T2 data, others only T0 and T1), analyses were conducted using an available-case approach. The median patient age was 65 years (IQR, 56–74 years), with 57.0% (n = 53) were male. Among the 93 patients, 65 (69.9%) and 67 (72.8%) had ischemic stroke and hypertension, respectively. The median duration from stroke onset to T0 was 14 days (IQR, 12–18 days). Table 1 presents the clinicodemographic patient characteristics.

**Table 1:** Clinicodemographic patient characteristics (n = 93).

Characteristic	Value
**Age, yr**	65 (56–74)
**Sex (male:female)**	53 (57.0%):40 (43.0%)
**Stroke type (ischemic:hemorrhagic)**	65 (69.9%):28 (30.1%)
**Duration from stroke onset to T0 (transfer to the rehabilitation department after the completion of acute stroke care), d**	14 (12–18)
**Previous stroke**	12 (13.0%)
**BDNF genotype (Val/Val:Val/Met:Met/Met)**	23 (24.7%):44 (47.3%):26 (28.0%)
**Smoking**	16 (17.4%)
**Alcohol**	40 (43.5%)
**Comorbidities**	
**Hypertension**	67 (72.8%)
**Diabetes mellitus**	35 (38.0%)
**Dementia**	2 (2.2%)
**Rheumatic disorder**	5 (5.43%)
**Medication during comprehensive rehabilitation**	
**Selective serotonin reuptake inhibitors**	85 (92.4%)
**Acetylcholinesterase inhibitors**	37 (40.2%)
**Oral steroids**	8 (8.7%)
**rTMS during comprehensive rehabilitation**	23 (25.0%)

Continuous variables are expressed as median (IQR) or mean ± standard deviation, while categorical variables are presented as n (%).

BDNF, brain-derived neurotrophic factor; rTMS, repetitive transcranial magnetic stimulation

### Longitudinal changes in potential serum biomarker levels and functions

**Fig 2:**
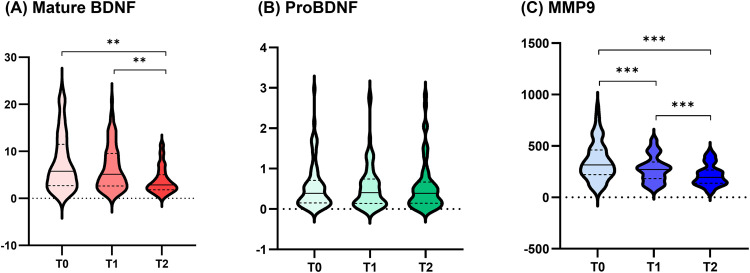
Serial levels of serum biomarkers in total patients. ^**^p < 0.01, ^***^p < 0.001 between each time point after Bonferroni correction. Serial changes in serum concentrations of (A) mature BDNF, (B) proBDNF, and **(C)** MMP-9. Abbreviations: BDNF, brain-derived neurotrophic factor; MMP-9, matrix metalloproteinase-9; T0, completion of acute stroke care; T1, 2 weeks after transfer to the rehabilitation department; T2, 3 months post-stroke onset.

Fig 2 demonstrates the serial changes in serum biomarker levels in the total population. Mature BDNF levels were significantly decreased from T0 to T2 and from T1 to T2 (T0 vs T1, p = 0.06; T0 vs T2, p = 0.003; T1 vs T2, p = 0.003), whereas proBDNF levels showed no significant changes (all p > 0.05). Serum MMP-9 levels were significantly decreased starting from T1 (T0 vs T1, p < 0.001; T0 vs T2, p < 0.001; T1 vs T2, p < 0.001). The serial changes in biomarker levels by total population and BNDF genotype group are detailed in Table 2. Among the 93 patients, 23 (24.7%), 44 (47.3%), and 26 (28.0%) patients had GG (Val/Val), GA (Val/Met), and AA (Met/Met) genotypes, respectively. In the unadjusted analysis, there were no significant among-group differences in the BDNF and proBDNF levels at each time point. However, in the analysis adjusted for the covariates sex, age, initial stroke severity, and stroke type, significant group differences in serum MMP-9 levels at T0 were observed (adjusted *p* < 0.05).

**Table 2:** Serial trends in serum biomarker levels in the total population and BDNF genotype groups.

Biomarker	Timepoint	Total (n = 93)	GG group (n = 23)	GA group (n = 44)	AA group (n = 26)	p ^a^	p ^b^
Mature BDNF	T0	5.78 [2.77–11.08]	6.36 [3.52–12.57]	5.71 [2.63–10.96]	5.40 [2.84–9.86]	0.89	0.19
	T1	5.11 [2.66–9.54]	4.79 [2.29–10.57]	5.16 [2.56–9.86]	5.08 [3.47–7.44]	0.96	0.21
	T2	2.84 [1.83–5.03]	3.17 [2.19–6.08]	2.81 [1.92–4.22]	2.62 [1.66–4.82]	0.71	0.57
ProBDNF	T0	0.39 [0.16–0.70]	0.34 [0.10–0.67]	0.40 [0.21–0.69]	0.41 [0.12–1.06]	0.64	0.75
	T1	0.40 [0.18–0.78]	0.29 [0.04–0.68]	0.36 [0.17–0.68]	0.47 [0.22–1.10]	0.29	0.84
	T2	0.39 [0.15–0.66]	0.23 [0.14–0.47]	0.39 [0.18–0.66]	0.52 [0.14–1.10]	0.63	0.46
MMP-9	T0	315.40 [222.50–458.50]	289.65 [225.40–377.90]	287.45 [199.10–461.10]	443.20 [274.35–543.55]	0.11	0.049*
	T1	271.15 [184.80–343.10]	271.15 [161.75–302.85]	249.40 [153.20–371.60]	290.20 [239.40–393.95]	0.23	0.05
	T2	193.60 [139.60–262.30]	249.95 [178.95–294.15]	183.85 [131.60–243.80]	190.60 [137.25–261.55]	0.20	0.43

* p < 0.05.

**p**^a^: ANOVA for between-group comparison of continuous variables.

**p**^b^: ANCOVA test with covariates (sex, age, NIHSS T0, stroke type) for between-group comparison.

Continuous variables are expressed as the median (IQR) or mean ± standard deviation.

Abbreviations: BDNF, brain-derived neurotrophic factor; MMP-9, matrix metalloproteinase-9; T0, completion of acute stroke care; T1, 2 weeks after transfer to the rehabilitation department; T2, 3 months post-stroke onset.

**Fig 3:**
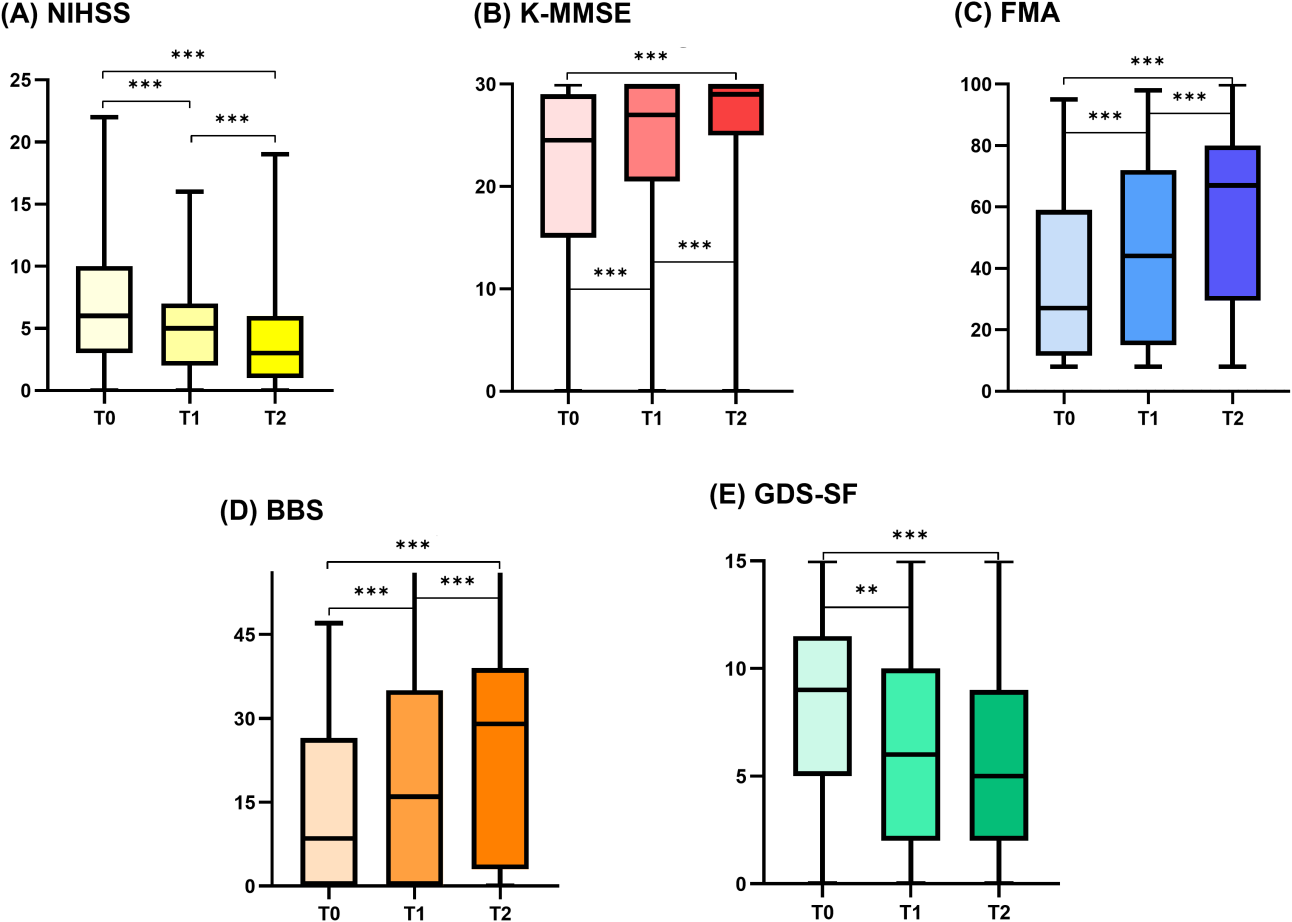
Serial functional status in total patients. ^*^p < 0.05, ^**^p < 0.01, ^***^p < 0.001 between each time point after Bonferroni correction. Serial changes in (A) stroke severity (NIHSS score), (B) cognitive function (MMSE score), (C) motor function (FMA score), (D) balance (BBS score), and depressive mood (GDS-SF score). Abbreviation: NIHSS, National Institutes of Health stroke scale; BBS, Berg Balance Scale; FMA, Fugl-Meyer Assessment; K-MMSE, Korean Mini-Mental State Examination; GDS-SF, Geriatric Depression Scale-Short Form; T0, completion of acute stroke care; T1, 2 weeks after transfer to rehabilitation department; T2, 3 months post-stroke onset.

Changes in functional status across time points are summarized in Fig 3. NIHSS scores consistently decreased starting from T0 (all p < 0.001). Furthermore, BBS, K-MMSE, and FMA scores gradually improved at each time point (T0 vs T1 p < 0.001, T0 vs T2 p < 0.001, T1 vs T2 p < 0.001). Meanwhile, although GDS-SF scores significantly improved from T0 to T1 (p = 0.02), no further changes between T1 and T2 were observed.

### Prognostic factors of functional status at the early subacute phase of stroke

The results of the univariate and multivariate analysis of baseline parameters (T0) associated with functional status at the early subacute phase (T1) are described in S2 and S3 Tables, respectively. In the univariate analysis, age, BDNF genotype, and NIHSS score at T0 were significantly associated with functional outcomes at T1. Multivariate regression analysis confirmed that older age (β = 0.03 and p = 0.04) and high NIHSS scores at T0 (β = 0.767 and p < 0.001) were independently associated with functional outcomes at T1. Age, BDNF genotype, baseline mature BDNF level, baseline proBDNF level, and BBS score at T0 showed significant associations in the univariate regression analysis. However, only a high BBS score at T0 was an independent influencing factor of balance function at T1 (β = 1.241 and p < 0.001). Further, multivariate regression analysis of K-MMSE, FMA, and GDS-SF scores at T1 suggested that high K-MMSE, FMA, and GDS-SF scores at T0 were independently related to improved cognitive function, motor impairment, and depressive mood at T1, respectively (all p < 0.001, Table 3). No other prognostic factors for these functional domains were identified.

**Table 3:** Multiple linear regression analysis of potential baseline (T0) parameters prognostic of functional status at the early subacute phase of stroke (T1).

Predictor	Unstandardized coefficient		t	p-Value	R^2^
	B	Std. error			
**NIHSS**					0.813
**(Constant)**	−1.983	0.925	−2.143		
**Age**	0.030	0.014	2.094	0.039^*^	
**Previous stroke, yes**	−0.362	0.634	−0.570	0.570	
**Number of Met allele = 1**	0.267	0.526	0.508	0.613	
**Number of Met allele = 2**	0.464	0.598	0.776	0.440	
**NIHSS at T0**	0.767	0.045	17.179	<0.001^***^	
**K-MMSE**					0.905
**(Constant)**	6.599	1.842	3.582		
**Age**	−0.035	0.024	−1.460	0.148	
**K-MMSE at T0**	0.888	0.033	26.640	<0.001^***^	
**FMA**					0.855
**(Constant)**	8.643	2.007	4.306		
**FMA at T0**	1.044	0.046	22.816	<0.001^***^	
**BBS**					0.841
**(Constant)**	8.380	6.893	1.216		
**Age**	−0.097	0.096	−1.007	0.321	
**Number of Met allele = 1**	−0.134	3.286	−0.041	0.968	
**Number of Met allele = 2**	−0.914	3.858	−0.237	0.814	
**Mature BDNF at T0**	0.368	0.276	1.334	0.191	
**ProBDNF at T0**	2.270	2.714	0.836	0.409	
**BBS at T0**	1.241	0.100	12.402	<0.001^***^	
**GDS-SF**					0.500
**(Constant)**	−3.094	1.905	−1.625		
**Age**	0.054	0.029	1.836	0.071	
**GDS-SF at T0**	0.787	0.107	7.369	<0.001^***^	

* p < 0.05, ** p < 0.01, *** p < 0.001 for the multivariate linear regression model.

NIHSS, National Institutes of Health Stroke Scale; K-MMSE, Korean Mini-Mental State Examination; FMA, Fugl-Meyer Assessment; BBS, Berg Balance Scale; BDNF, Brain-Derived Neurotrophic Factor; GDS-SF, Geriatric Depression Scale short form; T0, completion of acute stroke care; T1, 2 weeks after comprehensive rehabilitation

### Prognostic factors of functional status at the late subacute phase of stroke

S3 and S4 Tables show the results of the univariate and multivariate analyses of baseline factors prognostic of functional status at T2. Older age (β = 0.048 and p = 0.030), longer duration from stroke onset to completion of acute stroke care (β = 0.087 and p = 0.047), and higher baseline NIHSS score (β = 0.573 and p < 0.001) were independently associated with worse stroke severity at the late subacute stage. Meanwhile, hemorrhagic stroke type (β = 4.253 and p < 0.001) and higher K-MMSE score at baseline (β = 0.697 and p < 0.001) were associated with better cognitive function. T2 FMA scores were negatively related to age (β = −0.335 and p *=* 0.005) and were positively related to baseline FMA score (β = 0.891 and p < 0.001). Higher baseline BBS and GDS-SF scores were independently related to improved balance function and depressive mood at the late subacute phase, respectively (both p < 0.001, Table 4). There were no other prognostic factors for these two functional domains. In an exploratory analysis stratified by baseline serum mature BDNF level (high vs. low), no significant differences were observed in changes of neurological severity, motor function, balance, cognition, or depressive mood from T0 to T2 (S4 Table).

**Table 4:** Multiple linear regression analysis of potential baseline parameters (T0) prognostic of functional status at the late subacute phase of stroke (T2).

Predictor	Unstandardized coefficient		t	p-Value	R^2^
	B	Std. error			
**NIHSS score**					0.503
**(Constant)**	−3.964	1.632	−2.429		
**Age**	0.048	0.022	2.225	0.029^*^	
**Duration from stroke onset to T0**	0.087	0.043	2.018	0.047^*^	
**NIHSS score at T0**	0.573	0.077	7.484	<0.001^***^	
**K-MMSE score**					0.768
**(Constant)**	11.625	3.245	3.582		
**Age**	−0.037	0.034	−1.081	0.283	
**Stroke type, hemorrhagic**	4.253	1.012	4.203	<0.001^***^	
**Duration from stroke onset to T0**	−0.040	0.064	−0.631	0.530	
**K-MMSE score at T0**	0.694	0.052	13.411	<0.001^***^	
**FMA score**					0.710
**(Constant)**	44.473	7.631	5.828		
**Age**	−0.335	0.114	−2.927	0.005^**^	
**FMA score at T0**	0.891	0.066	13.422	<0.001^***^	
**BBS score**					0.636
**(Constant)**	19.930	9.917	2.010		
**Age**	−0.166	0.138	−1.205	0.235	
**Number of Met allele = 1**	3.531	4.917	0.718	0.477	
**Number of Met allele = 2**	−0.035	5.770	−0.006	0.995	
**Mature BDNF level at T0**	0.385	0.359	1.072	0.290	
**BBS score at T0**	1.136	0.146	7.806	<0.001^***^	
**GDS-SF score**					0.338
**(Constant)**	−0.335	2.037	−0.164		
**Age**	0.024	0.033	0.739	0.463	
**Previous stroke, yes**	0.870	1.365	0.637	0.526	
**GDS score at T0**	0.564	0.112	5.011	<0.001^***^	

NIHSS, National Institutes of Health Stroke Scale; K-MMSE, Korean Mini-Mental State Examination; FMA, Fugl-Meyer Assessment; BBS, Berg Balance Scale; BDNF, brain-derived neurotrophic factor; GDS-SF, Geriatric Depression Scale short form; T0, completion of acute stroke care; T2, 3 months post-stroke onset

## Discussion

The longitudinal design of this study, conducted under structured inpatient rehabilitation, offers a unique perspective on the biological underpinnings of recovery. Rather than serving as static prognostic markers, temporal changes in BDNF and MMP-9 may better represent ongoing neuroplastic responses during rehabilitation. This study shows that serum mature BDNF levels are significantly decreased as patients with stroke transition into the late subacute phase, whereas MMP-9 levels gradually decline from the early subacute phase to the late subacute phase. However, serum BDNF levels are not related to functional outcomes in both the early and late subacute phases. Overall, serum mature BDNF, proBDNF, and MMP-9 levels did not demonstrate independent prognostic values for functional status in the subacute phases. Meanwhile, BDNF genotypes do not influence functional status.

BDNF is released by neurons and peripheral tissues in response to injury or stress, such as in neurodegenerative diseases and traumatic brain injury [15,16]. BDNF exerts neuroprotective effects, including anti-apoptotic, anti-neurotoxic, anti-inflammatory, and neural regeneration effects, by regulating related cytokines and regenerative signaling [16]. Our results can theoretically be related to the release of BDNF from the brain and peripheral cells, as neurons are destroyed in the early stages of the disease, with levels gradually returning to baseline. Similar findings have been reported in previous studies [6].

The baseline serum levels of mature BDNF, proBDNF, and MMP-9 were not prognostic of functional status in the subacute phase of stroke in the current study. However, previous research has shown conflicting findings of either beneficial or unfavorable effects of BDNF levels on functional outcomes in stroke. In a previous analysis adjusted for age, sex, stroke severity, stroke classification, and stroke history, serum BDNF levels on day 5 were significantly associated with functional dependence [17]. In contrast, a large-scale prospective study involving more than 300 patients with stroke showed that serum BDNF levels had low predictive value for motor recovery, with a low area under the curve [18]. Further, one study reported a negative correlation between serum BDNF levels and stroke severity scores [19]. Conversely, another study reported a significant negative nonlinear cubic regression between BDNF concentration and stroke severity [20]. Lopez-Cancio et al.[21] also found no association between serum BDNF levels and functional outcomes over a 3-month follow-up in 83 patients with stroke. This discrepancy across studies may partly reflect the heterogeneity of ischemic stroke subtypes. In particular, acute ischemic small vessel disease is characterized by distinct pathophysiological mechanisms and recovery patterns [22], and future studies are warranted to determine whether the present biomarker trajectories are consistent across different ischemic stroke subtypes.

In addition, heterogeneity in rehabilitation modalities during the later subacute phase should be considered. After completion of standardized inpatient rehabilitation, patients transitioned to either outpatient rehabilitation programs or home-based exercise, which may have introduced variability in rehabilitation intensity and adherence. This variability could have influenced functional recovery trajectories and potentially attenuated associations between biomarker dynamics and clinical outcomes between T1 and T2. Similarly, the current study findings indicate a lack of significant association between serum BDNF levels and functional outcomes

Biochemically, ProBDNF and mature BDNF interact closely, with each protein playing an opposing role in brain function. proBDNF generally promotes apoptosis, while mature BDNF supports cell survival and plasticity [23]. Moreover, proBDNF is cleaved to generate a prodomain and mature BDNF. This process is active in both intracellular and extracellular spaces, and extracellular cleavage is regulated by MMP-9 [24]. MMP-9 is a zinc-dependent protease that degrades the extracellular matrix and basal lamina; its expression is upregulated following cerebral ischemia [25]. Both animal model and human studies have indicated a time-dependent expression of MMPs post-ischemia, contributing to brain edema and blood-brain barrier disruption [26,27]. From a biological perspective, the observed dissociation between the temporal decline in mature BDNF and MMP-9 levels and the relative stability of proBDNF levels in the present study may be interpreted as follows. Mature BDNF and MMP-9 may be transiently elevated in response to acute brain injury, followed by a gradual return toward baseline levels as injury-related pathophysiological processes resolve. In contrast, the relative stability of proBDNF levels suggests that precursor availability is preserved regardless of disease course. In this context, longitudinal changes in circulating mature BDNF may be more appropriately interpreted as reflecting evolving neurobiological states during rehabilitation rather than serving as direct determinants of functional recovery.

Further, baseline MMP-9 levels differed significantly according to the BDNF genotype in the adjusted analysis. Patients with the AA genotype exhibited the highest baseline serum MMP-9 levels. BDNF polymorphism significantly reduces the conversion of proBDNF to mature BDNF, both within and outside the cell, resulting in proBDNF being the primary secreted form [28]. However, the current study found no significant differences in serum levels of mature BDNF or proBDNF among the genotypes across all assessment timepoints. These findings suggest that differences in MMP-9 concentration may be associated with the initial inflammatory process rather than with extracellular cleavage. Most biomarker mechanistic studies have been conducted in animal models; thus, the findings may not fully translate to human physiology.

The finding that BDNF genotype did not independently influence functional status differs from what has been suggested by prior literature. These may also reflect population-specific differences in BDNF genotype distribution. In Western and Middle Eastern populations, the GG genotype is typically dominant (≈60–76%), whereas the AA genotype is rare (≈2–3%); in contrast, our cohort showed a substantially higher prevalence of A-allele carriers (75.2%), consistent with reports from Asian populations [29–31]. Previous studies in Asian cohorts have reported no clear association between serum BDNF levels and BDNF genotype, suggesting that population-specific regulatory mechanisms may modulate mature BDNF availability despite the Val66Met polymorphism [32,33]. These discrepancies may partially explain the ambiguous results of this study. Further research involving a larger cohort of human patients is required to clarify these associations.

This study has limitations. The study may have been subject to two main types of bias. First, with a sample size of 93, the study population may not fully represent the broader population, raising concerns regarding external validity and constituting a sampling bias. Another limitation is the pattern of differential follow-up across the study period. Although 100 patients initially consented, only 93 completed T0, 90 completed T1, and 66 completed T2 assessments. Because different patients contributed data at different time points, analyses were conducted using an available-case approach rather than a traditional intention-to-treat framework. This may introduce imbalance across time points and reduce the statistical power of longitudinal comparisons. In addition, future studies employing linear mixed-effects models may better account for within-subject variability and missing data across repeated measurements, potentially providing more robust estimates of longitudinal biomarker–outcome relationships. Also, the IQR method was used to remove various outliers during serum protein level measurements using ELISA. Although this is a commonly applied approach, unintended loss of meaningful data may have occurred owing to the lack of recommended normal ranges for each serum biomarker. Particularly, proBDNF had the lowest values among the three proteins, and minor decimal differences might have led to its exclusion. Additionally, the reduction in sample size owing to outlier removal may have decreased the statistical power of the analysis. In addition, although baseline brain images were obtained as part of routine acute stroke care, standardized quantitative imaging parameters suitable for research-level analysis were not prospectively collected and therefore could not be incorporated into the present analyses. The last key limitation of this study is the absence of a healthy control group. As a result, the biological meaning of the observed trajectories should be interpreted with caution. Future studies including matched healthy controls will be necessary to clarify the reference values and potential clinical relevance of these biomarkers.

Despite these limitations, this study is valuable as a human-based research that collects serial data, including not only of three biochemically interconnected biomarkers (mature BDNF, proBDNF, and MMP-9), but also of various clinical and functional levels, from the subacute to chronic phases of stroke. Although the biomarkers did not independently predict functional outcomes, their dynamic changes across the subacute period align with known neuroplastic and inflammatory processes after stroke. These findings imply that serum BDNF and MMP-9 may serve as biological indicators of neural adaptation, rather than prognostic determinants per se.

In conclusion, serum levels of mature BDNF, proBDNF, and MMP-9 were not predictive of functional status in patients with stroke. However, mature BDNF and MMP-9 levels significantly change according to the disease course, the clinical implications of these biomarker dynamics remain uncertain. Further studies with larger cohorts and appropriate control groups are required to clarify whether these biomarkers hold any reliable prognostic or mechanistic value in stroke recovery.

## Supporting information

S1 Table Number of missing data after data cleaning for serum biomarkers. (DOCX)

S2 Table Univariate linear regression analysis of potential baseline parameters (T0) prognostic of functional status at the early subacute phase of stroke (T1). (DOCX)

S3 Table Univariate linear regression analysis of potential baseline parameters (T0) prognostic of functional status at the late subacute phase of stroke (T2). (DOCX)

S4 Table Comparison of functional changes from T0 to T2 according to baseline mature BDNF level (high vs. low). (DOCX)
