# Supplementary material for: Serum BDNF levels as a potential prognostic marker for functional recovery in stroke: Preliminary findings from a prospective observational study
Source: PLoS One. 2026 Feb 27;21(2):e0343929. doi: 10.1371/journal.pone.0343929 (PMC12948131; doi:10.1371/journal.pone.0343929)
Supplement: S1 Table — (DOCX) [file pone.0343929.s001.docx]

**S1 Table.**Number of missing data after data cleaning for serum biomarkers

| Biomarker | | Before cleaning | After cleaning | Removed |
| --- | --- | --- | --- | --- |
| Mature BDNF | T0 | 93 | 91 | 2 |
|  | T1 | 90 | 86 | 4 |
|  | T2 | 66 | 61 | 5 |
| ProBDNF | T0 | 93 | 79 | 14 |
|  | T1 | 90 | 76 | 14 |
|  | T2 | 66 | 57 | 9 |
| MMP-9 | T0 | 93 | 87 | 6 |
|  | T1 | 90 | 86 | 4 |
|  | T2 | 66 | 65 | 1 |

BDNF, brain-derived neurotrophic factor; MMP-9, matrix metalloproteinase-9; T0, completion of acute stroke care; T1, 2 weeks after comprehensive rehabilitation; T2, 3 months post-stroke onset
