# Supplementary material for: Serum BDNF levels as a potential prognostic marker for functional recovery in stroke: Preliminary findings from a prospective observational study
Source: PLoS One. 2026 Feb 27;21(2):e0343929. doi: 10.1371/journal.pone.0343929 (PMC12948131; doi:10.1371/journal.pone.0343929)
Supplement: S2 Table — (DOCX) [file pone.0343929.s002.docx]

**S2 Table.** Univariate linear regression analysis of potential baseline parameters (T0) prognostic of functional status at the early subacute phase of stroke (T1)

| Predictor | Unstandardized coefficient | | t | p-Value | R^2^ |
| --- | --- | --- | --- | --- | --- |
|  | B | Std. error |  |  |  |
| NIHSS score at T1 |  |  |  |  |  |
| Age | 0.066 | 0.030 | 2.210 | 0.030^*^ | 0.053 |
| Sex (male: 0, female: 1) | 0.422 | 0.924 | 0.457 | 0.649 | 0.002 |
| Stroke type (ischemic: 0, hemorrhagic: 1) | -0.265 | 1.000 | -0.264 | 0.792 | 0.001 |
| Duration from stroke onset to T0 | 0.065 | 0.059 | 1.085 | 0.281 | 0.013 |
| Previous stroke (yes: 1, no: 0) | 1.737 | 1.343 | 1.293 | 0.199^#^ | 0.019 |
| Number of Met alleles | 1.847 | 1.101 | 1.678 | 0.097^#^ | 0.079 |
| Mature BDNF levels at T0 | -0.066 | 0.079 | -0.838 | 0.405 | 0.008 |
| ProBDNF levels at T0 | -0.334 | 0.828 | -0.404 | 0.688 | 0.002 |
| MMP-9 levels at T0 | 0.002 | 0.003 | 0.848 | 0.399 | 0.009 |
| NIHSS levels at T0 | 0.787 | 0.042 | 18.617 | <0.001^***^ | 0.799 |
| K-MMSE score at T1 |  |  |  |  |  |
| Age | -0.225 | 0.069 | -3.260 | 0.002^**^ | 0.109 |
| Sex (male: 0, female: 1) | -2.695 | 2.187 | -1.232 | 0.221 | 0.017 |
| Stroke type (ischemic: 0, hemorrhagic: 1) | 2.953 | 2.353 | 1.255 | 0.213 | 0.018 |
| Duration from stroke onset to T0 | -0.150 | 0.141 | -1.062 | 0.291 | 0.013 |
| Previous stroke (yes: 1, no: 0) | -2.943 | 3.192 | -0.922 | 0.359 | 0.010 |
| Number of Met alleles | -2.348 | 2.642 | -0.889 | 0.377 | 0.060 |
| Mature BDNF levels at T0 | -0.032 | 0.191 | -0.167 | 0.868 | <0.001 |
| ProBDNF levels at T0 | 1.930 | 2.076 | 0.929 | 0.356 | 0.012 |
| MMP-9 levels at T0 | -0.003 | 0.006 | -0.550 | 0.584 | 0.004 |
| K-MMSE score at T0 | 0.903 | 0.032 | 28.181 | <0.001^***^ | 0.902 |
| FMA score at T1 |  |  |  |  |  |
| Age | -0.257 | 0.206 | -1.248 | 0.215 | 0.017 |
| Sex (male: 0, female: 1) | -3.291 | 6.245 | -0.527 | 0.600 | 0.003 |
| Stroke type (ischemic: 0, hemorrhagic: 1) | -4.296 | 6.748 | -0.637 | 0.526 | 0.005 |
| Duration from stroke onset to T0 | -0.298 | 0.403 | -0.739 | 0.462 | 0.006 |
| Previous stroke (yes: 1, no: 0) | -4.663 | 9.161 | -0.509 | 0.612 | 0.003 |
| Number of Met alleles | -8.577 | 7.603 | -1.128 | 0.262 | 0.038 |
| Mature BDNF levels at T0 | 0.568 | 0.531 | 1.071 | 0.287 | 0.013 |
| ProBDNF levels at T0 | 0.473 | 5.826 | 0.081 | 0.935 | <0.001 |
| MMP-9 levels at T0 | -0.010 | 0.017 | -0.589 | 0.557 | 0.004 |
| FMA score at T0 | 1.044 | 0.046 | 22.816 | <0.001^***^ | 0.855 |
| BBS score at T1 |  |  |  |  |  |
| Age | -0.331 | 0.159 | -2.081 | 0.042^*^ | 0.066 |
| Sex (male: 0, female: 1) | 0.064 | 5.043 | 0.013 | 0.990 | <0.001 |
| Stroke type (ischemic: 0, hemorrhagic: 1) | 0.141 | 5.410 | 0.026 | 0.979 | <0.001 |
| Duration from stroke onset to T0 | -0.093 | 0.300 | -0.309 | 0.759 | 0.002 |
| Previous stroke (yes: 1, no: 0) | -3.222 | 7.111 | -0.453 | 0.652 | 0.003 |
| Number of Met alleles | -12.295 | 6.015 | -2.044 | 0.045^*^ | 0.067 |
| Mature BDNF levels at T0 | 0.803 | 0.427 | 1.881 | 0.065^#^ | 0.056 |
| ProBDNF levels at T0 | 8.050 | 4.336 | 1.856 | 0.069^#^ | 0.063 |
| MMP-9 levels at T0 | -0.011 | 0.014 | -0.819 | 0.417 | 0.012 |
| BBS score at T0 | 1.280 | 0.081 | 15.831 | <0.001^***^ | 0.837 |
| GDS-SF score at T1 |  |  |  |  |  |
| Age | 0.078 | 0.037 | 2.125 | 0.037^*^ | 0.057 |
| Sex (male: 0, female: 1) | 0.071 | 1.130 | 0.063 | 0.950 | <0.001 |
| Stroke type (ischemic: 0, hemorrhagic: 1) | 0.321 | 1.172 | 0.274 | 0.785 | 0.001 |
| Duration from stroke onset to T0 | 0.070 | 0.078 | 0.902 | 0.370 | 0.011 |
| Previous stroke (yes: 1, no: 0) | 2.118 | 1.643 | 1.290 | 0.201 | 0.022 |
| Number of Met alleles | 0.025 | 1.341 | 0.019 | 0.985 | <0.001 |
| Mature BDNF levels at T0 | -0.003 | 0.093 | -0.032 | 0.975 | <0.001 |
| ProBDNF levels at T0 | -0.707 | 0.991 | -0.713 | 0.478 | 0.008 |
| MMP-9 levels at T0 | -0.001 | 0.003 | -0.218 | 0.828 | 0.001 |
| GDS-SF score at T0 | 0.816 | 0.108 | 7.589 | <0.001^***^ | 0.474 |

^#^p<0.2, ^*^p<0.05, ^**^p<0.01, ^***^p<0.01 for the univariate linear regression model

BDNF, brain-derived neurotrophic factor; MMP9, matrix metalloproteinase-9; T0, completion of acute stroke care; T1, 2 weeks after transfer to the rehabilitation department; NIHSS, National Institutes of Health Stroke Scale; K-MMSE, Korean Mini-Mental State Examination; FMA, Fugl-Meyer Assessment; BBS, Berg Balance Scale; GDS-SF, Geriatric Depression Scale-Short Form
