# Supplementary material for: Serum BDNF levels as a potential prognostic marker for functional recovery in stroke: Preliminary findings from a prospective observational study
Source: PLoS One. 2026 Feb 27;21(2):e0343929. doi: 10.1371/journal.pone.0343929 (PMC12948131; doi:10.1371/journal.pone.0343929)
Supplement: S3 Table — (DOCX) [file pone.0343929.s003.docx]

**S3 Table.** Univariate linear regression analysis of potential baseline parameters (T0) prognostic of functional status at the late subacute phase of stroke (T2)

| Predictor | Unstandardized coefficient | | t | p-Value | R^2^ |
| --- | --- | --- | --- | --- | --- |
|  | B | Std. error |  |  |  |
| NIHSS score at T2 |  |  |  |  |  |
| Age | 0.052 | 0.029 | 1.810 | 0.074^#^ | 0.040 |
| Sex (male: 0, female: 1) | 0.575 | 0.882 | 0.652 | 0.516 | 0.005 |
| Stroke type (ischemic: 0, hemorrhagic: 1) | 0.413 | 0.932 | 0.443 | 0.659 | 0.003 |
| Duration from stroke onset to T0 | 0.137 | 0.056 | 2.462 | 0.016^**^ | 0.072 |
| Previous stroke (yes: 1, no: 0) | -0.527 | 1.388 | -0.380 | 0.705 | 0.002 |
| Number of Met alleles | 0.931 | 1.050 | 0.887 | 0.378 | 0.011 |
| Mature BDNF levels at T0 | -0.066 | 0.077 | -0.857 | 0.394 | 0.010 |
| ProBDNF levels at T0 | 0.154 | 0.836 | 0.184 | 0.855 | 0.001 |
| MMP-9 levels at T0 | -0.001 | 0.002 | -0.518 | 0.606 | 0.004 |
| NIHSS score at T0 | 0.618 | 0.077 | 8.040 | <0.001^***^ | 0.453 |
| K-MMSE score at T2 |  |  |  |  |  |
| Age | -0.185 | 0.060 | -3.102 | 0.003^**^ | 0.111 |
| Sex (male: 0, female: 1) | -2.089 | 1.853 | -1.127 | 0.263 | 0.016 |
| Stroke type (ischemic: 0, hemorrhagic: 1) | 3.845 | 1.919 | 2.003 | 0.049^*^ | 0.050 |
| Duration from stroke onset to T0 | -0.197 | 0.121 | -1.631 | 0.107^#^ | 0.033 |
| Previous stroke (yes: 1, no: 0) | 0.232 | 2.927 | 0.079 | 0.937 | <0.001 |
| Number of Met alleles | -2.559 | 2.199 | -1.164 | 0.248 | 0.020 |
| Mature BDNF levels at T0 | -0.010 | 0.163 | -0.063 | 0.950 | <0.001 |
| ProBDNF levels at T0 | 1.258 | 1.733 | 0.726 | 0.470 | 0.008 |
| MMP-9 levels at T0 | 0.001 | 0.005 | 0.184 | 0.855 | 0.001 |
| K-MMSE score at T0 | 0.706 | 0.053 | 13.266 | <0.001^***^ | 0.698 |
| FMA score at T2 |  |  |  |  |  |
| Age | -0.335 | 0.208 | -1.613 | 0.111^#^ | 0.032 |
| Sex (male: 0, female: 1) | -2.991 | 6.294 | -0.475 | 0.636 | 0.003 |
| Stroke type (ischemic: 0, hemorrhagic: 1) | -0.101 | 6.652 | -0.015 | 0.988 | <0.001 |
| Duration from stroke onset to T0 | -0.277 | 0.410 | -0.676 | 0.501 | 0.006 |
| Previous stroke (yes: 1, no: 0) | 3.683 | 9.924 | 0.371 | 0.712 | 0.002 |
| Number of Met alleles | -7.427 | 7.470 | -0.994 | 0.323 | 0.015 |
| Mature BDNF levels at T0 | 0.453 | 0.544 | 0.833 | 0.408 | 0.009 |
| ProBDNF levels at T0 | 0.334 | 6.008 | 0.056 | 0.956 | <0.001 |
| MMP-9 levels at T0 | 0.006 | 0.017 | 0.356 | 0.723 | 0.002 |
| FMA score at T0 | 0.891 | 0.069 | 12.815 | <0.001^***^ | 0.678 |
| BBS score at T2 |  |  |  |  |  |
| Age | -0.252 | 0.170 | -1.484 | 0.143^#^ | 0.035 |
| Sex (male: 0, female: 1) | 1.739 | 5.298 | 0.328 | 0.744 | 0.002 |
| Stroke type (ischemic: 0, hemorrhagic: 1) | -0.310 | 5.653 | -0.055 | 0.957 | <0.001 |
| Duration from stroke onset to T0 | -0.238 | 0.337 | -0.708 | 0.482 | 0.008 |
| Previous stroke (yes: 1, no: 0) | 0.282 | 7.866 | 0.036 | 0.972 | <0.001 |
| Number of Met alleles | -12.215 | 6.298 | -1.939 | 0.057^#^ | 0.070 |
| Mature BDNF levels at T0 | 0.867 | 0.450 | 1.926 | 0.059^#^ | 0.058 |
| ProBDNF levels at T0 | 3.067 | 4.825 | 0.636 | 0.528 | 0.008 |
| MMP-9 levels at T0 | -0.018 | 0.014 | -1.296 | 0.200 | 0.029 |
| BBS score at T0 | 1.146 | 0.138 | 8.312 | <0.001^***^ | 0.600 |
| GDS-SF score at T2 |  |  |  |  |  |
| Age | 0.053 | 0.034 | 1.560 | 0.123^*^ | 0.034 |
| Sex (male: 0, female: 1) | 0.073 | 1.034 | 0.071 | 0.944 | <0.001 |
| Stroke type (ischemic: 0, hemorrhagic: 1) | 0.500 | 1.054 | 0.474 | 0.637 | 0.003 |
| Duration from stroke onset to T0 | 0.081 | 0.072 | 1.125 | 0.265 | 0.018 |
| Previous stroke (yes: 1, no: 0) | 2.168 | 1.522 | 1.424 | 0.159^#^ | 0.029 |
| Number of Met alleles | 0.632 | 1.214 | 0.520 | 0.605 | 0.006 |
| Mature BDNF levels at T0 | 0.064 | 0.089 | 0.717 | 0.476 | 0.008 |
| ProBDNF levels at T0 | -0.349 | 0.907 | -0.385 | 0.702 | 0.003 |
| MMP-9 levels at T0 | 0.000 | 0.003 | 0.137 | 0.891 | <0.001 |
| GDS-SF score at T0 | 0.591 | 0.108 | 5.446 | <0.001^***^ | 0.324 |

^#^p<0.2, ^*^p<0.05, ^**^p< 0.01, ^***^p<0.001 for the univariate linear regression model

BDNF, brain-derived neurotrophic factor; MMP9, Matrix Metalloproteinase-9; T0, completion of acute stroke care; T2, 3 months post-stroke onset; NIHSS, National Institutes of Health Stroke Scale; K-MMSE, Korean Mini-Mental State Examination; FMA, Fugl-Meyer Assessment; BBS, Berg Balance Scale; GDS-SF, Geriatric Depression Scale-Short Form
