# Supplementary material for: Serum BDNF levels as a potential prognostic marker for functional recovery in stroke: Preliminary findings from a prospective observational study
Source: PLoS One. 2026 Feb 27;21(2):e0343929. doi: 10.1371/journal.pone.0343929 (PMC12948131; doi:10.1371/journal.pone.0343929)
Supplement: S4 Table — (DOCX) [file pone.0343929.s004.docx]

**S4 Table.** Comparison of functional changes from T0 to T3 according to baseline mature BDNF level (high vs. low)

| Outcome (ΔT2–T0) | BDNF Low group | BDNF High group | p-value |
| --- | --- | --- | --- |
| NIHSS | −2.0 (−4.0 to −0.5) | −2.0 (−3.0 to −0.5) | 0.37 |
| FMA upper | 10 (5.0 to 18.0) | 4 (0.0 to 13.0) | 0.12 |
| BBS | 16.0 (0.0 to 24.0) | 16.0 (6.0 to 28.0) | 0.46 |
| GDS-SF | −1.0 (−4.25 to 0.25) | −1.0 (−4.5 to 0.0) | 0.94 |
| K-MMSE | 1.0 (0.0 to 4.0) | 1.5 (0.0 to 4.0) | 0.84 |

Values are presented as median (interquartile range).
P-values were calculated using the Wilcoxon rank-sum test.
Baseline serum mature BDNF levels were dichotomized into high and low groups using a median split

BDNF, brain-derived neurotrophic factor; T0, completion of acute stroke care; T2, 3 months post-stroke onset; NIHSS, National Institutes of Health Stroke Scale; K-MMSE, Korean Mini-Mental State Examination; FMA, Fugl-Meyer Assessment; BBS, Berg Balance Scale; GDS-SF, Geriatric Depression Scale-Short Form
